# Sounds of the Heart

**Published:** 1858-07

**Authors:** 


*Sounds of the Heart.*—In some recent papers published by Dr. Halford, in the London Medical Times and Gazette, some conclusive experiments are given, showing that the sounds are produced exclusively by the tension of the valves, the first by that of the auriculo-ventricular, the second by that of the semilunar valve.

